# Supplementary material for: Identification and functional analysis of non-coding regulatory small RNA FenSr3 in Bacillus amyloliquefaciens LPB-18
Source: PeerJ. 2023 May 15;11:e15236. doi: 10.7717/peerj.15236 (PMC10194069; doi:10.7717/peerj.15236)
Supplement: Supplemental Information 4 [file peerj-11-15236-s004.zip › KO/CK-vs-T1_map/map04975.html]

KEGG PATHWAY: Fat digestion and absorption - Reference pathway


|  |  |
| --- | --- |
| **Fat digestion and absorption - Reference pathway** |  |

[
Pathway menu
| Organism menu
| Pathway entry
| Show description
| User data mapping
]

Fat is an important energy source from food. More than 95% of dietary fat is long-chain triacylglycerols (TAG), the remaining being phospholipids (4.5%) and sterols. In the small intestine lumen, dietary TAG is hydrolyzed to fatty acids (FA) and monoacylglycerols (MAG) by pancreatic lipase. These products are then emulsified with the help of phospholipids (PL) and bile acids (BA) present in bile to form micelles. Free FAs and MAGs are taken up by the enterocyte where they are rapidly resynthesized in endoplasmic reticulum (ER) to form TAG. PLs from the diet as well as bile - mainly LPA - too are absorbed by the enterocyte and are acylated to form phosphatidic acid (PA), which is also converted into TAG. Absorbed cholesterol (CL) is acylated to cholesterol esters (CE). Within the ER, TAG joins CE and apolipoprotein B (ApoB) to form chylomicrons that enter circulation through the lymph.

|  |  |  |
| --- | --- | --- |
| Reference pathway | 184% 150% 122% 100% 82% 67% 55% | 图片下载 |
